# Linking the severity of illness and the weekend effect: a cohort study examining emergency department visits

**DOI:** 10.1186/s13049-018-0542-x

**Published:** 2018-09-05

**Authors:** Iben Duvald, Anders Moellekaer, Mathias A. Boysen, Betina Vest-Hansen

**Affiliations:** 1 Interdisciplinary Centre for Organizational Architecture, Department of Management, Business and Social Sciences, Aarhus University, Fuglesangs Allé 4, build. 2610-336, 8210 Aarhus V, Denmark; 2 DESIGN EM – Research Network for Organizational Design and Emergency Medicine, Fuglesangs Allé 4, build. 2610, 8210 Aarhus V, Denmark; 3 Department of Business Development and Technology, Business and Social Sciences, Aarhus University, Birk Centerpark 15, 7400 Herning, Denmark; 4 Research Center for Emergency Medicine, Department of Clininal Medicine, Aarhus University and Department of Emergency Medicine, Aarhus University Hospital, Aarhus, Denmark; 5 Enversion A/S, Fiskerivej 12, 8000 Aarhus C, Denmark

**Keywords:** Weekend effect, Patient characteristics, 30-day mortality, Disease severity, Triage, Length of stay, Emergency department

## Abstract

**Background:**

Despite extensive research on the “weekend effect” i.e., the increased mortality associated with hospital admission during weekend, knowledge about disease severity in previous studies is limited. The aim of this study is to examine patient characteristics, including disease severity, 30-day mortality, and length of stay (LOS), according to time of admission to an emergency department.

**Methods:**

Our study encompassed all patients admitted to a Danish emergency department in 2014–2015. Using data from electronic patient records, this study examines patient characteristics including age, gender, Charlson Comorbidity Index score, triage score, and primary diagnosis. Triage score and transfer to intensive care unit (ICU) were used as indicators of disease severity. LOS within the department and within the hospital was examined. Age- and sex-standardized 30-day mortality rates comparing patients with the same triage score admitted at daytime, evening, and nighttime on weekdays and on weekends were computed. To test differences, a Cox regression analysis was added.

**Results:**

We included 35,459 patient visits, of which 10,435 (32%) started on a weekend. There were no large differences in baseline characteristics between patients admitted on weekdays and those admitted on weekends. The relative risk (RR) for being triaged orange or red was 1.16 (95% confidence interval (CI) 1.06–1.28, *P =* 0.0017) for weekend admissions as compared with weekday admissions. Weekend admissions were twice as likely as weekday admissions to be transferred to the ICU (RR, 1.96; 95% CI 1.53–2.52, *P =* 0.0000). No significant changes were found in LOS. The 30-day mortality rate increased with disease severity regardless of time of admission. When comparing the 30-day mortality rate for patients with the same triage score, the trend was toward a higher mortality when admission occurred during the weekend. Increasing mortality rate was significant for patients admitted at evening on weekends with a hazard ratio of 1.32 (95% CI 1.03–1.70, *P* = 0.027) when compared with patients admitted on daytime on weekdays.

**Conclusions:**

When comparing weekday and weekend admissions, the 30-day mortality rate increased for patients admitted at evening on weekends after adjusting for comorbidity and triage score, indicating that the weekend effect was independent of changes in illness severity.

**Electronic supplementary material:**

The online version of this article (10.1186/s13049-018-0542-x) contains supplementary material, which is available to authorized users.

## Background

Today, most acute patients are admitted to hospital with an initial stay in the emergency department. A reorganization of the Danish health care system and the establishment of 21 emergency departments has directed about 70% of acute patients to an emergency department [1]. Many patients remain in the emergency department and are discharged without in-hospital admission. Due to an aging population and an increasing number of patients with chronic conditions, the number of patients admitted to hospital, and therefore treated within emergency departments, is increasing [2].

A large number of national and international studies have documented a difference in mortality rate for acute patients admitted to hospital on the weekend compared to those admitted on a weekday. This phenomenon is also called the weekend effect [3–10]. The extent to which this effect reflects the health care provided or the characteristics of the admitted patients is unclear.

A concern that has been prominent in the earlier literature is whether changes in patient characteristics e.g., disease severity may be an explanation of the weekend effect. Some studies points that patients admitted on weekends might be sicker than those admitted on weekdays, while other studies conclude that “the findings were probably not due to unmeasured factors such as the severity of illness” [3]. Early studies were criticized for not considering disease severity variation [11]. However, the studies have mainly used administrative data, which contain limited information on disease severity. Thus, rigorous analysis using databases with clinical information on the patients is needed.

In recent years, electronic patient records (EPR) have been recognized as a rich data source when examining differences in patient outcomes. Several studies of the weekend effect use data from EPR [12, 13]. One UK study, using EPR data to investigate 30-day mortality among emergency admissions on weekdays and on weekends, investigated the effect of 15 commonly measured blood test results, and found some association between the test results and the increased mortality rate for weekend admissions [13]. Thus, the use of data from the EPR can be important in the search for explanations of the difference in mortality for patients admitted during weekdays and weekends.

The weekend effect is well documented among acutely hospitalized patients, and emergency department admissions in Canada, the United States, UK, and other European countries [14–16]. However, there has been limited research on the weekend effect for acute patients admitted to Scandinavian emergency departments, including Danish emergency departments. One study, examining the medical records of 5385 patients admitted to a Danish emergency department during a three-month period, found that patients attending on weekends had indications of increased 30-day mortality compared with patients attending on weekdays. The study was able to adjust for disease severity based on clinical information from EPR [12].

To address these gaps in the literature, we conducted a cohort study of all patients admitted to a Danish emergency department during daytime, evening, or nighttime in a two-year period (2014–2015). By using administrative data and clinical data from the EPR, we examined patient characteristics including age, gender, Charlson Comorbidity Index (CCI) score, primary diagnoses, triage score, transfers to intensive care unit (ICU), and length of stay (LOS) of weekend versus weekday admissions. In addition, we calculated and compared the age- and sex-standardized 30-day mortality for patients clustered in six different triage groups (red, orange, yellow, green, blue, and patients without a triage score). Triage data are widely used in emergency departments to evaluate disease severity [17]. By using the EPR data, we are able to get data about patients’ triage scores. To our knowledge, the triage score, i.e., indicator for disease severity, has not been used before as stratification variable in studies comparing mortality rates for acute patients admitted on weekdays and on weekends. To test differences within mortality rates for weekday and weekend admissions and to adjust for possible confounders, a Cox proportional hazards regression was performed.

## Methods

### Study design and setting

We conducted a cohort study. We identified all admissions to the emergency department at Viborg Regional Hospital, Regional Hospital Central Jutland, between 1 January 2014 and 31 December 2015 using administrative data from the EPR.

In Denmark, a free, tax-funded health care system ensures that all citizens have unrestricted access to general practitioners (GPs) and hospital care [18, 19]. Every Danish citizen is affiliated with a GP who in daytime refers the patient to the hospital. In evening, at nighttime, and on weekends, GPs rotate staffing of regional out-of-hours service centers, where they receive all patient calls. In case of a life-threatening condition or injury, patients can dial 1–1-2 and present by ambulance to the emergency department.

The Emergency Department at Viborg Regional Hospital is one of five emergency hospitals in Central Denmark Region. The emergency department employs 145 nurses and physicians, including eight senior physicians. With backing from physicians from other departments, all acute patients with a referral diagnosis covering general surgery, orthopedic surgery, and internal medicine are diagnosed and treated in the emergency department. Children, other than those with minor injuries, are received in the pediatric department, and patients with serious heart-related events bypass the emergency department are directed to the department of cardiology. Patients with psychiatric diseases are admitted to a psychiatric hospital. The emergency department, Viborg Regional Hospital, has previously been described in detail [20].

### Data source

We have used data from MidtEPJ, the EPR developed by Systematic and used by all somatic and psychiatric hospitals in Central Denmark Region. Viborg Regional Hospital, as a part of Regional Hospital Central Jutland, was one of the last hospitals in the region to implement MidtEPJ in 2013. The MidtEPJ is a work, communication and documentation tool. It is accessible by multiple authorized users (e.g., nurses, physicians, and secretaries) and supports the clinical workflow across groups of health professionals, departments, and hospitals. The MidtEPJ documents patient morbidity, treatment, and care over time. It contains both administrative data on hospital admission, including date (hours and minutes), department, source of admission, and clinical data such as age, gender, primary and secondary diagnoses, and triage score. The MidtEPJ is linked to the unique Civil Personal Registration number (CPR number) every Danish citizen is assigned at birth and to residents upon immigration. The CPR number is a 10-digit number that contains embedded information on birth date and sex. The CPR number ensures unambiguous patient identification. When using MidtEPJ data for research, the data is extracted directly from the source system itself and is stored in a regional data warehouse (named the Business Intelligence Portal). The data warehouse gathers data from a number of the region’s different electronic systems, including MidtEPJ for quality assurance and health statistics purposes.

To compute the CCI score for each patient, data were obtained from the Danish National Patient Registry (DNPR), which is a central medical registry that has recorded information on all hospital admissions since 1995 [21]. The record of each admission is linked to the CPR number.

Information on all-cause mortality within 30 days following the admission date was captured by linking the patient’s CPR number to the Danish Civil Registration System (CRS). Established in 1968, the CRS stores complete and daily updated information on vital events, and can be retrieved for research purposes while protecting the Danish citizens’ anonymity [22]. CRS thus contains complete information on vital events of all patients included in this study. Patients were followed from date of admission until the date of death from any causes, the 30th day after discharge, or emigration, whichever occurred first.

### Study population

We included all admissions to the emergency department between 1 January 2014 and 31 December 2015 (a flowchart for the patient visits included in the study is available in see Additional file 1: Appendix I). This time interval was chosen because the emergency department became an independent department (independent leadership, budget, unique administrative department code) on 1 January 2014. The emergency department started receiving a wider range of patient categories, and the emergency room, formerly a part of the department of orthopedic surgery, became a part of the emergency department. On 1 January 2016 the organization of the emergency department changed again, as the department began to receive more medical patients who earlier would have been admitted to a nearby hospital.

To ensure complete follow-up, we only included patients with a CPR number.

A patient’s visit at the hospital may consist of admissions to one or more departments. A patient admitted to the emergency department may be transferred to the ICU and afterward transferred to an internal medical department. During one hospital visit, some patients may be admitted to the same department more than once. In this study, we included hospital visits with up to five consecutive admissions. Furthermore, if more than four hours elapsed between two admissions, we considered it as two different hospital visits.

We excluded those patients treated at two clinics, that are organizationally part of the emergency department but are physically located in the cities of Skive and Silkeborg. Moreover, we excluded patients with missing information about date of finishing treatment within the emergency department. For the 30-day mortality analysis, we excluded nine patients due to invalid date of death.

### Time of admission

In this study, the exposure was the time of admission. We defined six time periods: daytime (from 7:00 a.m. to 2:59 p.m.), evening (from 3:00 p.m. to 10:59 p.m.), and nighttime (from 11:00 p.m. to 6:59 a.m.) on weekdays and on weekends. Patients were considered weekend admissions if they were admitted between 3:00 p.m. on Friday and 6:59 a.m. on Monday. Patients admitted on all other days and times were considered weekday admissions. Public holidays were coded as weekend. We chose the time periods based on knowledge about how the emergency department was staffed and organized on weekdays and on weekends [20]. Classifying time of admission into six periods, including daytime, evening, and nighttime on weekdays and on weekends, is an attempt to provide a more subtle description of the weekend effect.

### Explanatory variables

For each of the six time periods we described age, gender, comorbidity, triage score, and source of admission (GP, other hospital departments, self-referral, or other). Patient age was described based on five groups: 0–19, 20–39, 40–59, 60–79, and > 80. The department (and hospital) the patients were transferred to after initial treatment within the emergency department, as well as primary diagnosis reflecting the reason for admission and identified at the time of discharge, was examined too. According to Danish guidelines and the guidelines of World Health Organization, the primary diagnosis assigned at hospital discharge should be the main reason for a patient’s hospitalization [23]. We coded the diagnoses according to the International Classification of Diseases, 10th revision (ICD-10). However, we combined the infectious diseases in one group and merged other non-infectious diseases into a single diagnostic group, leaving us with fourteen diagnostic groups (details are outlined in see Additional file 2: Appendix II).

The CCI score was computed for each patient. This index reflects the number and seriousness of comorbid diseases. In this study, we collected data based on admissions recorded within the 10 years prior to admission. Three groups were created: Low (index score 0), Moderate (index score 1–2) and High (index score > =3), categorical based on ICD-10 codes.

As a proxy for disease severity, we included the triage score. All emergency departments in Central Denmark Region use the tool Danish Emergency Process Triage system (DEPT). DEPT is a five-step triage system that prioritizes patients according to the degree of life or truancy threat and thereby is indicative of how fast they are to be seen by a physician. It is based on triage using vital signs (airway, oxygen saturation, respiratory rate, pulse, blood pressure, Glasgow Coma Score, and temperature), which are collected by nurses as an integral part of the initial process of care, combined with pre-defined attention points related to the symptoms the patient had when admitted. The DEPT score is categorized by five groups of triage scores: blue (minor injury, only used in the emergency room), green (not urgent), yellow (less urgent), orange (urgent) and red (life-threatening).

### Outcomes

For consistency with previous studies of the weekend effect, the main outcome in this study was 30-day mortality [10, 12]. LOS was secondary outcome. Emergency department and hospital LOS was calculated as the number of minutes from admission to the emergency department to final discharge or transfer to another hospital department from the emergency department. During the two-year study period, several patients had multiple emergency department visits. In the analysis of 30-day mortality, we included the last admission to the emergency department for each patient. In total, 21,736 patient-visits were included.

### Statistical analysis

We calculated the proportions of patients admitted during daytime, evening, and nighttime on weekdays and on weekends and characterized them according to patient characteristics. For each time group, we collected data on age, gender, CCI score, source of admission, primary diagnosis, triage score, transfer to another department, and LOS. Relative risks (RRs) for being triaged orange or red, for having a stay longer than 24 h within the emergency department, and for being transferred to ICU, comparing weekday admissions with weekend admission, were calculated. RRs were displayed with their 95% confidence intervals (CIs) and *P* values to indicate precision of estimate.

To facilitate comparisons, direct standardization adjusted for age and gender was used to compute the 30-day mortality rate for patients admitted to the emergency department in each of the six time periods (daytime, evening, and nighttime on weekdays and on weekends) [24]. As standard population for the mortality analysis, we applied the patients admitted in daytime on weekdays. Thus, for each time period, we estimated what would have been the 30-day mortality rate in this time period, if the population in that particular time period was equal according to age and gender with the one in our standard population. Mortality rates were displayed with their 95% confidence intervals (CIs) to indicate precision of the estimate. Subgroup analyses were performed for each triage score (red, orange, yellow, green and blue). Mortality rates for patients with missing triage score data were added. In our analysis, we used the triage score as stratification variable, because the triage score is used within the department as a proxy of severity of disease. Thus, the 30-day mortality rate may differ between these groups. More subgroup analyses were performed, comparing the mortality rates for patients discharged to home from the emergency department and the mortality rates for patients transferred to other departments after initial treatment within the emergency department.

A Cox proportional hazards regression with 95% confidence intervals [25, 26] was performed to test differences in the 30-day mortality rate for patients admitted to the emergency department in daytime, evening, and nighttime on weekdays and on weekends. As reference population, we applied the patients admitted in daytime on weekdays. To correct for differences in patient characteristics, we included confounders as following: age groups, gender, comorbidity burden, triage score, and whether the patients had or had not previous admissions to the emergency department within the two-year period included in the study. For the statistically tests, a *P* value less than 0.05 was considered significant.

Analyses were performed using the statistical software package STATA (version 11, Stata Corp, College Station, Texas, USA).

## Results

During the two-year study period, 35,459 visits were made to the emergency department, or about 49 visits per day. The total number of patients was 21,738, which means that many of the patients made several visits to the department. The median age of the patients was 48 years (IQR 25–70). Table 1 illustrates the demographic and clinical characteristics of the patients by time of admission. Patients admitted at nighttime were more frequently male, both on weekdays (52.8%) and on weekends (54.8%). There were no large differences in baseline characteristics between patients admitted on weekends and those admitted on weekdays. The percentage of patients admitted on weekends in the age group 20–39 was slightly higher than the percentage of patients in the same age group on weekdays. In total, 70.5% of the patients had a low CCI score, 19.6% had a moderate CCI score, and 9.9% had a high CCI score. As shown in Table 1, patients admitted during weekdays tended to have slightly higher CCI scores than patients admitted in the same time periods on weekends. This difference is most likely because the patients admitted on weekdays are slightly older than the patients admitted on weekends. In total, 75.7% of the patients were referred by the GP, 11.5% by other hospital departments, and 9.7% by others, while 3.1% of the patients were self-referrals. Most referrals from other hospital departments were during daytime, while most self-referrals presented during nighttime on weekends. Among the patients admitted at daytime or nighttime on weekdays the diagnosis “Factors influencing health status and contact with health services” were more prevalent, whereas patients admitted on weekends had a greater tendency to have diagnoses indicating injury or poisoning.

**Table 1:** Demographic and clinical characteristics by time of admission

	Weekday (From 7.00 am on Monday to 2.59 p.m. on Friday)			Weekend (From 3.00 p.m. on Friday to 6.59 a.m. on Monday)		
	Daytime (7.00 a.m. – 2.59 p.m.)	Evening (3.00 p.m. – 10.59 p.m.)	Nighttime (11.00 p.m. – 6.59 a.m.)	Daytime (7.00 a.m. – 2.59 p.m.)	Evening (3.00 p.m. – 10.59 p.m.)	Nighttime (11.00 p.m. – 6.59 a.m.)
	n (% of group)					
Overall	15,120 (42,6)	7185 (20,3)	1670 (4,7)	3566 (10,1)	5766 (16,3)	2152 (6,1)
Age groups						
0–19	2464 (16,3)	1558 (21,7)	189 (11,3)	543 (15,2)	1147 (19,9)	317 (14,7)
20–39	3007 (19,9)	1710 (23,8)	484 (29,0)	822 (23,0)	1442 (25,0)	692 (32,2)
40–59	3437 (22,7)	1505 (21,0)	370 (22,2)	774 (21,7)	1236 (21,4)	467 (21,7)
60–79	4061 (26,9)	1536 (21,4)	413 (24,7)	891 (25,0)	1217 (21,4)	451 (21,0)
> 80	2151 (14,2)	876 (12,2)	214 (12,8)	536 (15,0)	724 (12,6)	225 (10,5)
Gender						
Female	7549 (50,0)	3693 (51,4)	788 (47,2)	1769 (49,6)	2921 (50,7)	972 (45,2)
Male	7571 (50,1)	3492 (48,6)	882 (52,8)	1797 (50,4)	2845 (49,3)	1180 (54,8)
CCI score						
Low (0)	10,441 (69,1)	5162 (71,8)	1148 (68,7)	2478 (69,5)	4240 (73,5)	1541 (71,6)
Moderat (1–2)	3044 (20,1)	1382 (19,2)	337 (20,2)	730 (20,5)	1072 (18,6)	348 (17,8)
High (> = 3)	1635 (10,8)	641 (8,9)	185 (11,1)	358 (10,0)	454 (7,9)	227 (10,6)
Admission source						
GP	10,741 (71,0)	5875 (81,8)	1223 (73,2)	2791 (78,3)	4759 (82,5)	1437 (66,8)
Another hospital department	2663 (17,6)	489 (6,8)	89 (5,3)	403 (11,3)	357 (6,2)	87 (4,0)
No reference	512 (3,4)	197 (2,7)	32 (1,9)	82 (2,3)	150 (2,6)	133 (6,2)
Other	1200 (7,9)	621 (8,6)	326 (19,5)	290 (8,1)	499 (8,7)	495 (23,0)
Primary diagnosis						
Infectious diseases	1034 (6,8)	374 (5,2)	80 (4,8)	222 (6,2)	282 (4,9)	88 (4,1)
Neoplasm	10 (0,1)	5 (0,1)	1 (0,1)	3 (0,1)	1 (0,0)	0 (0)
Hematological diseases	162 (1,1)	43 (0,6)	2 (0,1)	18 (0,5)	18 (0,3)	0 (0)
Endocrine and nutritional diseases	136 (0,9)	107 (1,5)	20 (1,2)	28 (0,8)	60 (1,0)	19 (0,9)
Mental and behavioral disorders	91 (0,6)	99 (1,4)	36 (2,2)	22 (0,6)	75 (1,3)	101 (4,7)
Diseases of the nervous system	87 (0,6)	45 (0,6)	9 (0,5)	33 (0,9)	54 (0,9)	13 (0,6)
Diseases of the circulatory system	365 (2,4)	115 (1,6)	26 (1,6)	67 (1,9)	76 (1,3)	29 (1,4)
Diseases of the respiratory system	99 (0,7)	53 (0,7)	9 (0,5)	19 (0,5)	31 (0,5)	8 (0,4)
Diseases of the digestive system	388 (2,6)	130 (1,8)	48 (2,9)	92 (2,6)	127 (2,2)	60 (2,8)
Diseases of the musculoskeletal system	312 (2,1)	84 (1,2)	13 (0,8)	50 (1,4)	62 (1,0)	24 (1,1)
Diseases of the genitourinary system	179 (1,2)	63 (0,9)	21 (1,1)	59 (1,7)	54 (0,9)	25 (1,2)
Injury and poisoning	4038 (26,7)	2667 (37,1)	426 (25,5)	1231 (34,5)	2175 (37,7)	750 (34,9)
Factors influencing health status	6854 (45,3)	2738 (38,1)	727 (43,5)	1421 (39,9)	2232 (38,7)	758 (35,2)
Symptoms, signs, and abnormal findings	1276 (8,4)	615 (8,6)	239 (14,3)	265 (7,4)	461 (8,0)	256 (11,9)
Other	89 (0,6)	47 (0,7)	13 (0,8)	36 (1,0)	58 (1,0)	21 (1,0)

Overall, 32.4% of the patients were admitted on a weekend.

### Severity of disease

Table 2 portrays the triage score of patients admitted to the emergency department by time of admission. In total, 25.3% of the patients were triaged blue (24.6% on weekdays and 26.7% on weekends), 30.5% green (30.8% on weekdays and 29.9% on weekends), 12.2% yellow (11.7% on weekdays and 13.1% on weekends), 3.8% orange (3.6% on weekdays and 4.3% on weekends) and 1.7% red (1.6% on weekdays and 1.8% on weekends). The triage score of 26.6% of the patients admitted to the emergency department is unknown, because of missing data (characteristics of patients without a triage score are outlined in see Additional file 3: Appendix III). Throughout the day, there is a steady increase of patients triaged red, orange, or yellow, both on weekdays and on weekends. The only exception is patients who are triaged red and admitted in the evening. The RR for being triaged orange or red was 1.16 (95% CI 1.06–1.28, *P* value 0.0017) for weekend admissions as compared with weekday admissions.

**Table 2:** Triage color of patients admitted to the emergency department by time of admission

	Weekday (From 7.00 am on Monday to 2.59 p.m. on Friday)			Weekend (From 3.00 p.m. on Friday to 6.59 a.m. on Monday)		
	Daytime (7.00 a.m. – 2.59 p.m.)	Evening (3.00 p.m. – 10.59 p.m.)	Nighttime (11.00 p.m. – 6.59 a.m.)	Daytime (7.00 a.m. – 2.59 p.m.)	Evening (3.00 p.m. – 10.59 p.m.)	Nighttime (11.00 p.m. – 6.59 a.m.)
	n (% of group)					
Overall	15,120 (42,6)	7185 (20,3)	1670 (4,7)	3566 (10,1)	5766 (16,3)	2152 (6,1)
Triage 5 (blue, minor injury)	3588 (23,7)	2011 (28,0)	296 (17,7)	949 (26,6)	1646 (28,5)	468 (21,7)
Triage 4 (green, not urgent)	4756 (31,5)	2025 (28,2)	608 (36,4)	1034 (29,0)	1612 (28,0)	791 (36,8)
Triage 3 (yellow, less urgent)	1672 (11,1)	874 (12,2)	269 (16,1)	430 (12,1)	765 (13,3)	309 (14,4)
Triage 2 (orange, urgent)	486 (3,2)	271 (3,8)	103 (6,2)	129 (3,6)	254 (4,4)	108 (5,0)
Triage 1 (red, life threatening)	238 (1,6)	104 (1,4)	49 (2,9)	57 (1,6)	94 (1,6)	54 (2,5)
Triage missing	4380 (29,0)	1900 (26,4)	345 (20,7)	967 (27,1)	1395 (24,2)	422 (19,6)

While 66% of the patients admitted to the emergency department have been discharged to home, 34% (*n* = 12,147) have either been transferred to another part of the emergency department (*n* = 1132, 3%), to another department at the hospital (*n* = 10,619, 30%), or to another hospital (*n* = 396, 1%) (Table 3). Three quarters of the patients transferred to another department at the hospital were transferred to either medical departments (*n* = 2718, 26%), the orthopedic surgery department (*n* = 2175, 20%), or other surgical departments (*n* = 3037, 29%). More patients are transferred to other surgical departments at night both on weekdays and on weekends. The relatively large proportion of patients transferred to a neurological department at another hospital (24% of all patients transferred to a neurological department) is because thrombolysis is not performed at Viborg Regional Hospital, Regional Hospital Central Jutland.

**Table 3:** Number of patients transferred to other departments after initial treatment within the emergency department by time of admission

	Weekday (From 7.00 am on Monday to 2.59 p.m. on Friday)			Weekend (From 3.00 p.m. on Friday to 6.59 a.m. on Monday)		
	Daytime (7.00 a.m. – 2.59 p.m.)	Evening (3.00 p.m. – 10.59 p.m.)	Nighttime (11.00 p.m. – 6.59 a.m.)	Daytime (7.00 a.m. – 2.59 p.m.)	Evening (3.00 p.m. – 10.59 p.m.)	Nighttime (11.00 p.m. – 6.59 a.m.)
	n (% of group)					
Overall	5163 (42,5)	2379 (19,6)	674 (5,5)	1276 (10,5)	1971 (16,2)	684 (5,6)
Departments within the hospital						
Cardiology department	393 (7,6)	129 (5,4)	43 (6,4)	76 (6,0)	101 (5,1)	30 (4,4)
Neurological department	162 (3,1)	72 (3,0)	22 (3,3)	33 (2,6)	52 (2,6)	13 (1,9)
Other medical departments	1207 (23,4)	565 (23,8)	142 (21,1)	248 (19,4)	427 (21,7)	129 (18,9)
Orthopedic surgery department	790 (15,3)	432 (18,2)	132 (19,6)	300 (23,5)	390 (19,8)	131 (19,2)
Other surgical departments	1320 (25,6)	575 (24,2)	186 (27,6)	301 (23,6)	463 (23,5)	192 (28,1)
Gynecology department/obstetrics	241 (4,7)	136 (5,7)	37 (5,5)	83 (6,5)	108 (5,5)	24 (3,5)
Psychiatry	56 (1,1)	22 (0,9)	10 (1,5)	2 (0,2)	32 (1,6)	11 (1,6)
Pediatric ward	220 (4,3)	120 (5,0)	9 (1,3)	62 (4,9)	115 (5,8)	11 (1,6)
Intern transferring within the ED	565 (10,9)	205 (8,6)	46 (6,8)	95 (7,5)	153 (7,8)	68 (9,9)
ICU (intensive care unit)	47 (0,9)	58 (2,4)	31 (4,6)	27 (2,1)	52 (2,6)	49 (7,2)
Departments at other hospitals						
Cardiology department	6 (0,1)	0	0	1 (0,1)	4 (0,2)	1 (0,2)
Neurological department	47 (0,9)	18 (0,8)	6 (0,9)	15 (1,2)	24 (1,2)	4 (0,6)
Other medical departments	15 (0,3)	11 (0,5)	6 (0,9)	4 (0,3)	12 (0,6)	2 (0,3)
Orthopedic surgery department	20 (0,4)	2 (0,1)	0	8 (0,6)	0	0
Other surgical departments	52 (1,0)	19 (0,8)	1 (0,2)	16 (1,3)	23 (1,2)	12 (1,8)
Gynecology department/obstetrics	0	0	0	0	0	0
Psychiatry	1 (0,0)	7 (0,3)	2 (0,3)	0	2 (0,1)	4 (0,6)
Pediatric ward	1 (0,0)	0	0	0	0	0
Another ED	15 (0,3)	7 (0,3)	1 (0,2)	5 (0,4)	11 (0,6)	3 (0,4)
ICU (intensive care unit)	5 (0,1)	1 (0,0)	0	0	2 (0,1)	0

When comparing the proportion of patients transferred to the ICU from the emergency department in the different time periods, there is a steady increase in ICU admission rates throughout the day, both on weekdays and on weekends. On weekdays, the percentage increases from 0.9 in daytime to 4.6% at nighttime, while on weekends the percentage increases from 2.1 in daytime to 7.2% at nighttime. Nighttime, especially on weekends, was associated with the highest proportion of patients admitted to ICU. The RR for being transferred to the ICU was 1.96 (95% CI 1.53–2.52, *P* value 0.0000) for weekend admissions as compared with weekday admissions.

### Length of stay

The emergency department has a rule that patients who are expected to stay within the hospital more than 48 h, are transferred to other departments early during their admission. Only 1.5% of the patients have a LOS within the emergency department longer than 48 h. Table 4 shows LOS within the emergency department and within the hospital by time of admission.

**Table 4:** Patients’ LOS within the emergency department and within the hospital including their stay within the emergency department by time of admission

	Weekday (From 7.00 am on Monday to 2.59 p.m. on Friday)			Weekend (From 3.00 p.m. on Friday to 6.59 a.m. on Monday)		
	Daytime (7.00 a.m. – 2.59 p.m.)	Evening (3.00 p.m. – 10.59 p.m.)	Nighttime (11.00 p.m. – 6.59 a.m.)	Daytime (7.00 a.m. – 2.59 p.m.)	Evening (3.00 p.m. – 10.59 p.m.)	Nighttime (11.00 p.m. – 6.59 a.m.)
	n (% of group)					
Overall	15,120 (42,6)	7185 (20,3)	1670 (4,7)	3566 (10,1)	5766 (16,3)	2152 (6,1)
Length of stay within the emergency department						
0–59 min (< 1 h)	2320 (15,3)	998 (13,9)	225 (13,5)	597 (16,7)	942 (16,3)	379 (17,6)
60–179 min (1–2.59 h)	4520 (29,9)	2361 (32,9)	455 (27,3)	1114 (31,2)	1964 (34,1)	632 (29,4)
180–259 min (3–5.59 h)	3226 (21,3)	1385 (19,3)	147 (8,8)	683 (19,2)	1013 (17,6)	252 (11,7)
360–719 min (6–11.59 h)	2471 (16,3)	366 (5,1)	432 (25,9)	485 (13,6)	235 (4,1)	440 (20,5)
720–1439 min (12–23.59 h)	918 (6,1)	1696 (23,6)	331 (19,8)	222 (6,2)	1230 (21,3)	309 (14,4)
1440–2879 min (24–47.59 h)	1373 (9,1)	340 (4,7)	65 (3,9)	384 (10,8)	303 (5,3)	122 (5,7)
More than 2880 min (> 48 h)	292 (1,9)	39 (0,5)	15 (0,9)	81 (2,3)	79 (1,4)	18 (0,8)
Length of stay within the hospital						
0–59 min (< 1 h)	1864 (12,3)	797 (11,1)	154 (9,2)	486 (13,6)	738 (12,8)	265 (12,3)
60–179 min (1–2.59 h)	3622 (24,0)	1901 (26,5)	319 (19,1)	836 (23,4)	1529 (26,5)	485 (22,5)
180–259 min (3–5.59 h)	2272 (15,0)	945 (13,2)	92 (5,5)	442 (12,4)	659 (11,4)	190 (8,8)
360–719 min (6–11.59 h)	1407 (9,3)	223 (3,1)	267 (16,0)	272 (7,6)	136 (2,4)	362 (16,8)
720–1439 min (12–23.59 h)	711 (4,7)	1179 (16,4)	212 (12,7)	148 (4,2)	896 (15,5)	231 (10,7)
1440–2879 min (24–47.59 h)	1440 (9,5)	537 (7,5)	160 (9,6)	366 (10,3)	405 (7,0)	144 (6,7)
More than 2880 min (> 48 h)	3804 (25,2)	1603 (22,3)	466 (27,9)	1016 (28,5)	1403 (24,3)	475 (22,1)

Relative to weekdays, the percentage of patients who had a stay longer than 24 h within the emergency department on weekends increased in daytime from 11% to 13.0%, in evening from 5.3 to 6.6%, and at nighttime from 4.8 to 6.5%. However, the RR for having a stay longer than 24 h within the emergency department was 0.97 (95% CI 0.90–1.05, *P* value 0.4317) for weekend admissions as compared with weekday admissions.

Both on weekdays and on weekends, 40–50% of the patients, regardless of what time of the day they are admitted, are discharged to home from the emergency department or transferred to another department within 3 h.

### Mortality

In total, 664 patients (3.1%) died within 30 days after admission date. Table 5 shows the crude and age- and sex-standardized 30-day mortality rate for patients discharged from the emergency department and from another department, and for the six different triage groups. The age- and sex standardized 30-day mortality rate for patients admitted in daytime was 2.8% (95% CI 2.4–3.1) on weekdays and 3.2% (95% CI 2.5–3.9) on weekends. For patients admitted in the evening, the rate was 3.4% (95% CI 2.9–4.0) on weekdays and 3.7% (95% CI 3.1–4.4) on weekends, and for patients admitted in nighttime, the rate was 3.6% (95% CI 2.5–4.7) on weekdays and 4.5% (95% CI 3.3–5.6) on weekends.

**Table 5:** Crude and age- and sex standardized 30-day mortality rates for variation in discharge and for the six different triage colors among patients by time of admission to the emergency department

The patient’s last visit to the ED (21,736)	Weekday (From 7.00 am on Monday to 2.59 p.m. on Friday)					Weekend (From 3.00 p.m. on Friday to 6.59 a.m. on Monday)					
	Daytime (7.00 a.m. – 2.59 p.m.)	Evening (3.00 p.m. – 10.59 p.m.)		Nighttime (11.00 p.m. – 6.59 a.m.)		Daytime (7.00 a.m. – 2.59 p.m.)		Evening (3.00 p.m. – 10.59 p.m.)		Nighttime (11.00 p.m. – 6.59 a.m.)	
	Reference	Crude (%)	Adj. % (95% CI)	Crude (%)	Adj. % (95% CI)	Crude (%)	Adj. % (95% CI)	Crude (%)	Adj. % (95% CI)	Crude (%)	Adj. % (95% CI)
Overall	2.8 (2.4–3.1)	3.0	3.4 (2.9–4.0)	3.6	3.6 (2.5–4.7)	3.4	3.2 (2.5–3.9)	3.3	3.7 (3.1–4.4)	3.5	4.5 (3.3–5.6)
Discharge											
Discharged from the ED	0.9 (0.6–1.1)	1.0	1.2 (0.8–1.6)	1.5	1.7 (0.7–2.8)	1.2	1.1 (0.6–1.6)	0.9	1.0 (0.6–1.5)	1.5	2.1 (1.0–3.1)
Discharged from another department	7.1 (6.2–8.1)	7.9	7.9 (6.5–9.4)	7.1	6.8 (4.3–9.2)	8.5	7.8 (6.0–9.7)	9.0	9.3 (7.6–11.0)	9.0	9.2 (6.4–12.0)
Triage missing	1.8 (1.4–2.3)	2.5	2.7 (1.8–3.7)	5.1	5.3 (2.5–8.1)	3.5	2.2 (1.3–3.2)	3.1	3.1 (2.0–4.2)	4.6	5.8 (2.9–8.8)
Triage 5 (blue)	0.2 (0.0–0.4)	0.3	0.4 (0.0–0.7)	0	0 (0.0–0.0)	0.3	0.3 (0.0–0.7)	0	0 (0.0–0.0)	0.3	0.6 (0.0–1.7)
Triage 4 (green)	2.8 (2.2–3.4)	2.8	3.1 (2.1–4.1)	2.4	2.8 (1.0–4.6)	2.4	2.4 (1.2–3.6)	2.9	3.3 (2.1–4.4)	2.6	3.8 (1.8–5.7)
Triage 3 (yellow)	6.7 (5.1–8.4)	6.2	6.4 (4.3–8.5)	5.4	5.8 (2.1–9.6)	8.8	7.2 (4.2–10.2)	5.7	6.4 (4.0–8.8)	3.9	4.9 (1.4–8.3)
Triage 2 (orange)	15.0 (10.8–19.2)	15.4	18.6 (11.6–25.7)	8.9	8.5 (1.4–15.7)	20.0	21.3 (10.6–32.1)	18.1	19.7 (12.9–26.4)	11.3	19.3 (8.2–30.3)
Triage 1 (red)	20.5 (13.7–27.4)	18.5	22.0 (0-.)	16.0	20.8 (4.3–37.4)	12.5	9.4 (1.4–17.4)	33.3	39.3 (26.3–52.2)	29.2	37.6 (0-.)

Regardless of what time the patients were admitted to the emergency department, the mortality rate for patients who were transferred to another department after their initial treatment in the emergency department was higher than for patients discharged from the emergency department. When looking at the patients discharged to home from the emergency department, the age- and sex- standardized 30-day mortality rate for patients admitted on weekends compared to those admitted on weekdays were similar.

As expected, the triage score was directly associated with the 30-day mortality rate, which is lowest for patients triaged blue and highest for patients triaged red. Comparing the 30-day mortality rate for patients with the same triage score admitted in the same time periods on weekdays and on weekends, we found indications of increased mortality rate for patients admitted on weekends with an orange, or red triage score (Table 5), with exception of patients triaged red admitted in daytime on weekends. When comparing the patients triaged orange admitted on weekdays and on weekends, the age- and sex standardized 30-day mortality rate for patients admitted in daytime was 15.0% (95% CI 10.8–19.2) on weekdays and 21.3% (95% CI 10.6–32.1) on weekends. For patients admitted in the evening the rate was 18.6% (95% CI 11.6–25.7) on weekdays and 19.7% (95% CI 12.9–26.4) on weekends, and for patients admitted in nighttime, the rate was 8.5% (95% CI 1.4–15.7) on weekdays and 19.3% (95% CI 8.2–30.3) on weekends. Although these results were consistent, the findings were not statistically significant.

Due to the indication of a higher 30-day mortality rate for patients admitted on weekends with an orange or red triage score, a Cox regression analysis were added to test differences (Table 6). The analysis showed, increasing 30-day mortality rate to be significant for patients admitted in evening on weekends with a hazard ratio of 1.32 (95% CI 1.03–1.70) and a *P* value of 0.027, when compared with patients admitted on daytime on weekdays.

**Table 6:** Results of Cox proportional hazards regression analysis

	Number (N)	Mortality (%)	Crude mortality rate (95% CI)	Adj. mortality rate (95% CI)*	*P* value**
Weekday (From 7.00 am on Monday to 2.59 p.m. on Friday)					
Daytime (7.00 a.m. – 2.59 p.m.)	9257	2.8%	1 (reference)	1 (reference)	
Evening (3.00 p.m. – 10.59 p.m.)	4371	3.0%	1.08 (0.88–1.34)	1.20 (0.95–1.52)	0.131
Nighttime (11.00 p.m. – 6.59 a.m.)	972	3.5%	1.27 (0.89–1.82)	0.87 (0.57–1.32)	0.514
Weekend (From 3.00 p.m. on Friday to 6.59 a.m. on Monday)					
Daytime (7.00 a.m. – 2.59 p.m.)	2234	3.4%	1.25 (0.97–1.61)	1.08 (0.80–1.46)	0.603
Evening (3.00 p.m. – 10.59 p.m.)	3526	3.3%	1.20 (0.96–1.49)	1.32 (1.03–1.70)	0.027
Nighttime (11.00 p.m. – 6.59 a.m.)	1376	3.5%	1.27 (0.93–1.72)	1.29 (0.90–1.84)	0.169

*Adjusted for age groups, gender, comorbidity burden, triage score, and whether the patients had or had not previous admissions to the emergency department within the two-year period included in the study

**For the adjusted mortality rate

## Discussion

In this study of 35,459 visits to a Danish emergency department, we found that 32.4% of the patients were admitted on a weekend. There were no large differences in baseline characteristics between patients admitted on weekends and on weekdays. The 30-day mortality rate increased with disease severity, i.e., higher triage score, regardless of time of admission. When comparing the 30-day mortality rate for patients with the same triage score, the trend was toward a higher mortality rate for patients admitted during the weekend. By using a Cox regression analysis, we demonstrated that patients admitted via the emergency department at evening on weekends had an increased risk of dying within 30 days when compared with patients admitted at daytime on weekdays. This result was significant, and the increase in mortality persisted after adjusting for possible confounders; age, gender, comorbidity burden, triage score, and whether the patients had or had not previous admissions to the emergency department within the two-year period included in the study. Due to the choice of six time periods, this study shows both a daily variation and a variation between weekdays and weekends.

One possible explanation to the weekend effect, which have been discussed heavily in previous literature, is disease severity. Our results show that more patients admitted at weekends were triaged orange or red, and more patients were transferred to the ICU on weekends. However, we are aware that ICU transfers also depended on the availability of ICU beds. Vest-Hansen et al. found a similar result in their study of acute medical patients’ out-of-hours and weekend admissions to Danish medical departments [10]. However, the increase in 30-day mortality rate persisted after adjusting for both triage score and comorbidity burden. The differences within 30-day mortality rate found in this study may be due to changes within the health care provided as suggested by previous studies. The current study did not directly examine the organization and delivery of care on weekdays and on weekends. However, ethnographic fieldwork conducted in the same emergency department showed differences within the organization of the emergency department, weekdays and weekends, including a reduction in staffing and staffing experience, and changing working patterns [20]. Similar organizational differences have been observed in other Danish emergency departments [27].

As EPR systems become the norm in modern health care, it is natural to explore this treasure trove of data for improving health care and research. The key strength of this study is its use of data from the MidtEPJ, allowing us to analyze the disease severity using the data on triage score. Emergency departments use triage to determine the clinical priority of patients based on their presenting features in order to decrease morbidity and mortality. With the triage system, health care personnel can identify patients who need immediate attention, who can safely wait, or who may not need emergency care at all. However, using triage categories as indicators for disease severity has limitations. Only the initial triage color was used, and as described elsewhere, the triage score is sometimes changed, either because of changes in patient’s condition or due to organizational reasons such as lack of resources and time [20].

Another possible limitation is the sample size. When comparing the 30-day mortality rate for patients with the same triage score, the trend was toward a higher mortality rate for patients admitted during the weekend. However, the sample size of the study was not sufficient to show statistically significance in the analysis using the triage score as stratification variable. To test the differences within mortality rates for weekday and weekends admissions, a Cox regression analysis of all the patients were added. Statistically significance was reached, when comparing the 30-day mortality rate for patients admitted at evening on weekends with patients admitted in daytime on weekdays.

The quality of the data used from the large population-based databases is known to be accurate [28, 29], and both the data from the large population-based databases and the EPR-data were routinely collected independent of this study, thereby limiting certain types of bias. However, variations in practices is a known bias when using administrative data. Nurses collected the triage scores used in this study as an integral part of the initial process of care within the emergency department, and during an ethnographic fieldwork within the department, the first author observed variations in the nurses’ practices. The results of the fieldwork are described elsewhere [20]. However, we assume that coding practices is not likely to be a source of bias in our study, as the triage score would have to be systematically coded differently at the weekend compared with weekdays, and none of the nurses did only work at weekends. Moreover, some of the differences in registration practices resulting in missing data was avoided by the first author’s extensive knowledge of registration practices. One example was about how the nurses documented the blue triage score in the emergency room, which differed from the rest of the department. By being aware of this when extracting the data, we avoided a huge gap in data on triage score. However, the triage score of 26.6% of the patients admitted to the emergency department is still missing. The occurrence of missing data is well known when using secondary data sources for research [30]. In contrast to a previous study [12], we included patients without a triage score, but as an independent group. We expect that this group consists of patients either not ill (blue triage color) or critically ill. One explanation of the missing triage scores could be that in acute situations documentation in patient records is not always carried out due to logistics, prioritization, and treatment. Moreover, patients admitted to the emergency department as trauma patients do not have a regular triage and will appear as not being triaged.

The present study investigated one Danish emergency department and interpretations may not be generalizable to other settings. However, the cohort included all patients admitted to the emergency department during a two-year period. Thus, the population was a highly diverse patient group, including both medical, surgical and orthopedic surgical patients. The Danish health care system may differ from the systems in other countries. However, the results of this study support the evidence of a higher mortality for acute patients admitted on weekends when compared with admissions on weekdays [3–10]. This study extends this by grouping patients according to their triage score. One previous study has used the triage score as an indicator for disease severity, but only as a confounder [12]. Moreover, no studies of acute patients admitted to emergency departments have distinguished between daytime, evening, and nighttime on weekdays and on weekends, when comparing mortality rates, LOS, and patient characteristics including disease severity. This distinction is highly relevant when looking at how the Danish emergency departments, including the one at Viborg Regional Hospital, are organized [20, 27].

Also mentioned in a previous study, it is being admitted at weekends, rather than merely being in hospital at weekends, that has been consistently associated with higher mortality risk. Reduced weekend staffing and resources should affect all patients in hospital at weekends, not just those newly admitted [13]. However, within an emergency department, most patients only stay for a shorter while, which makes the admission time highly relevant when investigating the mortality rate for patients admitted to an emergency department on weekdays and on weekends. In this study, 40–50% of the patients admitted to the emergency department, regardless of what time of the day they were admitted, were discharged to home or transferred to another department within 3 h, and 91% within 24 h. Thus, only a few patients admitted on a weekday will be affected by how the emergency department is organized on weekends.

Death within 30 days of discharge from an emergency department is a rare event. During the two-year study period, only 13 patients triaged red and discharged from the emergency department died within 30 days of admission date. The reason for this is that critically ill patients often have a longer hospital stay, and after initial treatment in the emergency department, they are transferred to other departments. The results of this study confirm this by showing that the 30-day mortality rate was higher for patients transferred to and discharged from other departments. The differences within the 30-day mortality rate for patients admitted in the emergency department on weekdays and on weekends are still interesting, especially when the triage score of the patients is included. The triage is performed within the emergency department, and differences within this crucial and complex diagnostic and treatment phase within patients’ hospital stay may cause differences within the mortality rate. In this study, the 30-day mortality rate was also chosen, because of the possibilities to compare the results with other studies of the weekend effect. However, when analyzing the quality of treatment within emergency departments, the mortality rate may not be the most important parameter.

## Conclusion

In conclusion, when comparing acute patients’ admissions to an emergency department on weekdays and on weekends, the patient admitted at evening on weekends had an increased 30-day mortality rate when compared to patients admitted on daytime on weekdays. The reasons why is unknown. More patients were transferred to the ICU on weekends, and more patients were triaged red or orange, when they were admitted on weekends. However, the excess mortality rate for patients admitted at evening on weekends was found after adjusting for both triage score and comorbidity burden as possible confounders, indicating that the weekend effect was independent of changes in illness severity.

## Additional files

Additional file 1: **Appendix I.** Flowchart for all patient-visits to the emergency department in 2014–2015. (DOCX 28 kb)

Additional file 2: **Appendix II.** ICD-10 codes of each primary diagnostic group. (DOCX 20 kb)

Additional file 3: **Appendix III.** Characteristics for patients without triage score. (DOCX 17 kb)

## References

[1] Mattsson MS, Jørsboe H. Danske studier af akutte patientforløb efter opstart af fælles akutmodtagelser. (Danish studies of acute treatment after initiation of emergency departments). Ugeskr Laeger 2014;15:1396–1398. (In Danish). 25292231

[2] Sørup CM, Jacobsen P, Forberg JL. Evaluation of emergency department performance – a systematic review on recommended performance and quality-in-care measures. SJTREM. 2013;21(62):1–14. 10.1186/1757-7241-21-62 PMC3750595 23938117

[3] Bell CM, Redelmeier DA (2001). Mortality among patients admitted to hospitals on weekends compared with weekdays. N Engl J Med.

[4] Cram P, Hillis SL, Barnett M, Rosenthal GE (2004). Effects of weekend admission and hospital teaching status on in-hospital mortality. Am J Med.

[5] Barba R, Losa JE, Velasco M, Guijarro C, García de Casasola G, Zapatero A (2006). Mortality among adult patients admitted to the hospital on weekends. Eur Journal of Inter Med.

[6] Aylin PP, Yunus A, Bottle A, Majeed A, Bell D (2010). Weekend mortality for emergency admissions: a large multicentre study. Qual Saf Health Care.

[7] Mikulich O, Callaly E, Bennett K, O’Riordan D, Silke B (2011). The increased mortality associated with a weekend emergency admission is due to increased illness severity and altered case-mix. Acute Med.

[8] Sharp AL, Choi HJ, Hayward RA (2013). Don’t get sick on the weekend: an evaluation of the weekend effect on mortality for patients visiting US EDs. Am J Emerg Med.

[9] Ruiz M, Bottle A, Aylin PP (2015). The global comparators project: international comparison of 30-day in-hospital mortality by day of the week. BMJ Qual Saf.

[10] Vest-Hansen B, Riis AH, Sørensen HT, Christiansen CF (2015). Out-of-hours and weekend admissions to Danish medical departments: admission rates and 30-day mortality for 20 common medical conditions. BMJ Open.

[11] Lilford RJ, Chen YF (2015). The ubiquitous weekend effect: moving past proving it exists to clarifying what causes it. BMJ Qual Saf.

[12] Biering K, Nielsen RF, Pérez N (2016). Admission-time-dependent variation in mortality in a Danish emergency department. Dan Med J.

[13] Walker AS, Mason A, Quan TP, Fawcett NJ, Watkinson P, Llewelyn M, Stoesser N, Finney J, Davies J, Wyllie DH, Crook DW, Peto TEA (2017). Mortality risks associated with emergency admissions during weekends and public holidays: an analysis of electronic health records. Lancet.

[14] Webb M. The weekend effect: a rapid review of the literature. http://www2.nphs.wales.nhs.uk:8080/healthserviceqdtdocs.nsf/Main%20Frameset?OpenFrameSet&Frame=Right&Src=%2Fhealthserviceqdtdocs.nsf%2F61c1e930f9121fd080256f2a004937ed%2F7f113a4146140de1802578b1002d7781%3FOpenDocument%26AutoFramed (2011). Accessed 30 Sep 2017.

[15] De Cordova PB, Phibbs CS, Bartel AP, Stone PW. Twenty-four/seven: a mixed-method systematic review of the off-shift literature. J Adv Nurs. 2012:1454–68. 10.1111/j.1365-2648.2012.05976.x PMC3428734 22905343

[16] Zhou Y, Li W, Herath C, Xia J, Hu B, Song F, Cao S, Lu Z. Off-hour admission and mortality risk for 28 specific diseases: a systematic review and meta-analysis of 251 cohorts. J Am Heart Assoc. 2016;5 10.1161/JAHA.115.003102 PMC4943279 26994132

[17] Christ M, Grossmann F, Winter D, Bingisser R, Platz E (2010). Modern triage in the emergency department. Dtsch Arztebl Int.

[18] Pedersen KM, Christiansen T, Bech M (2005). The Danish health care system: evolution – not revolution - in a decentralized system. Health Econ.

[19] Christiansen T (2012). Ten years of structural reforms in Danish healthcare. Health Policy.

[20] Duvald I. Fits and misfits between the information processing requirements and capacities: the weekend effect in a hospital emergency department. 2018. Working paper.

[21] Schmidt M, Schmidt SAJ, Sandegaard JL, Ehrenstein V, Pedersen L, Sørensen HT (2015). The Danish National Patient Registry: a review of content, data quality and research potential. Clin Epidemiol.

[22] Schmidt M, Pedersen L, Sørensen HT (2014). The Danish civil registration system as a tool in epidemiology. Eur J Epidemiol.

[23] Vest-Hansen B, Riis AH, Sørensen HT, Christiansen CF (2014). Acute admissions to medical departments in Denmark: diagnoses and patient characteristics. European J Intern Med.

[24] Kirkwood BR, JAC S (2003). Essential medical statistics.

[25] Greenland S (1989). Modeling and variable selection in epidemiologic analysis. Am J Public Health.

[26] Hosmer DW, Lemeshow S, May S. Applied survival analysis: regression modeling of time to event data. 2nd ed. Hoboken, New Jersey: Wiley. 2008.

[27] Møllekær AB, Duvald I, Eskildsen J, Obel B, Madsen B, Kirkegaard H. The organisation of Danish emergency departments. Eur J Emer Med. 2018; [Epub ahead of print] 10.1097/MEJ.0000000000000554 29958243

[28] Sørensen HT, Baron JA, Olsen J, Saracci R, Trichopoulos D (2010). Registries and medical databases. Teaching epidemiology.

[29] Pedersen CB, Gotzsche H, Møller JO, Mortensen PB (2016). The Danish civil registration system. A cohort of eight million persons. Dan Med Bull.

[30] Sørensen HT, Sabroe S, Olsen J (1996). A framework for evaluation of secondary data sources for epidemiological research. Int J Epidemiol.

