# Supplementary material for: Linking the severity of illness and the weekend effect: a cohort study examining emergency department visits
Source: Scand J Trauma Resusc Emerg Med. 2018 Sep 5;26:72. doi: 10.1186/s13049-018-0542-x (PMC6125948; doi:10.1186/s13049-018-0542-x)
Supplement: Supplementary file 1 — Appendix I. Flowchart for all patient-visits to the emergency department in 2014–2015. (DOCX 28 kb) [file 13049_2018_542_MOESM1_ESM.docx]

**Additional file 1**

**Flowchart for all patient-visits to the emergency department in 2014-2015**

All admissions to the five emergency departments in the region in 2014-2015

237,302

All admissions to the emergency department, Viborg Regional Hospital, in 2014-2015

47,108 (19.9%)

All admissions to the emergency department, Viborg Regional Hospital, physically placed in Viborg, in 2014-2015

38,674

Exclusion (admissions to the two clinics placed in Skive and Silkeborg, which is part of the emergency department, Viborg)

8,434 (17.9%)

All first-time admissions to the emergency department, Viborg Regional Hospital, physically placed in Viborg, in 2014-2015

35,675

Exclusion (any admissions to the emergency department after first admission within the same hospital visit)

2,999 (7.8%)

All first-time admissions to the emergency department, Viborg Regional Hospital, physically placed in Viborg, in 2014-2015

35,459

Exclusion (admissions with missing information about time of completion of the patient’s treatment within the department)

216

Exclusion - only in the analysis of 30-day mortality (admissions with invalid date of death)

9
