# Supplementary material for: Linking the severity of illness and the weekend effect: a cohort study examining emergency department visits
Source: Scand J Trauma Resusc Emerg Med. 2018 Sep 5;26:72. doi: 10.1186/s13049-018-0542-x (PMC6125948; doi:10.1186/s13049-018-0542-x)
Supplement: Supplementary file 2 — Appendix II. ICD-10 codes of each primary diagnostic group. (DOCX 20 kb) [file 13049_2018_542_MOESM2_ESM.docx]

**Additional file 2**

**ICD-10 codes of each primary diagnostic group**

| Disease category | ICD-10 |
| --- | --- |
| Infectious diseases | A00-B99 |
| - Infections of the blood-forming organs | D73.3 |
| - Infections of the endocrine organs | E06.0, E06.9, E32.1 |
| - CNS infections | G00-G02, G04-G07 |
| - Infections of the eye, ear, and adnexa | H00, H01.0, H03-1, H04.0, H04.3, H05.0, H06.1, H10, H13.0-1, H15.0, H.19.1-2, H22.0, H32.0, H44.0-1, H60.0-1, H60.3, H62.0-3, H65.0-1, H66.0-4, H66.9, H67.1, H67.8, H68.0, H70.2, H73.0, H75.0, H94.0 |
| - Heart infections | I00-02, I30.1, I32.0-1, I33.0, I38, I39.8, I40.0, I41, I43.0, I52.0-1, I68.1, I98.1 |
| - Respiratory tract infections | J00-J06, J09-J18, J20-J22, J34.0, J36, J38.3D, J38.7G, J39.8A, J44.0, J85.1-3, J86 |
| - Infections of the digestive system | K04.0, K04.6-7, K05.2, K11.2-3, K12.2, K13.0A, K14.0A, K20.9A, K23.0-1, K35, K37, K57.0, K57.2, K57.4, K57.8, K61, K63.0, K65.0, K65.8I, K67, K75.0-1, K77.0, K80.0, K80.3-4, K81.0, K83.0, K85.9, K93.0-1 |
| - Skin and subcutaneous infections | L00-03, L05-08, L88 |
| - Infections of the musculo-skeletal system and connective tissue | M00-01, M46.1-5, M49.0-3, M60.0, M60.8, M63.0-2, M65.0-1, M68.0, M71.0-1, M86.0-2, M86.9, M90.0-2 |
| - Urinary tract infections | N10, N12, N13.6, N15.1, N16.0, N20.0I, N29.1, N30.0, N33.0, N34.0-1, N39.0 |
| - Male genital infections | N41, N43.1, N45.0, N45.9, N48.1-2, N49 |
| - Female genital infections | N61, N70-N77 |
| - Obstetrical infections | O23, O26.4, O41.1, O75.3, O85, O86, O88.3, O91, O98 |
| - Infectious complications of procedure, catheters etc. | T80.2, T81.4, T82.6-7, T83.5-6, T84.5-7, T85.7, T88.0, T89.9 |
| Neoplasms (Chapter II) | C00-D48 |
| Diseases of the blood and blood-forming organs and certain disorders involved in the immune system (Chapter III)* | D50-D89 |
| Endocrine, nutritional and metabolic disorders (Chapter IV)* | E00-E90 |
| Mental and behavioral disorders (Chapter V) | F00-F99 |
| Diseases of the nervous system (Chapter VI)* | G00-G99 |
| Diseases of the circulatory system (Chapter IX)* | I00-I99 |
| Diseases of the respiratory system (Chapter X)* | J00-J99 |
| Diseases of the digestive system (Chapter XI)* | K00-K93 |
| Diseases of the musculoskeletal system and connective tissue (Chapter XIII) | M00-M99 |
| Diseases of the genitourinary system (Chapter XIV)* | N00-N99 |
| Injury, poisoning and certain other consequences of external causes (Chapter XIX)* | S00-T98 |
| Factors influencing health status and contact with health services (Chapter XXI) | Z00-Z99 |
| Symptoms and abnormal findings, not elsewhere classified (Chapter XVIII) | R00-R99 |
| Other*  i.e., diseases of the eye and adnexa (Chapter VII)*, diseases of the ear and mastoid process (Chapter VIII)*, diseases of the skin and subcutaneous tissue (Chapter XII)*, diseases associated with pregnancy, childbirth and puerperium (Chapter XV)*, diseases originating in the perinatal period (Chapter XVI) and congenital malformations (Chapter XVII) | H00-H95, L00-L99, O00-O99, P00-P99, T00-T99 |

*except infectious diseases within the chapter
