# Supplementary material for: Linking the severity of illness and the weekend effect: a cohort study examining emergency department visits
Source: Scand J Trauma Resusc Emerg Med. 2018 Sep 5;26:72. doi: 10.1186/s13049-018-0542-x (PMC6125948; doi:10.1186/s13049-018-0542-x)
Supplement: Supplementary file 3 — Appendix III. Characteristics for patients without triage score. (DOCX 17 kb) [file 13049_2018_542_MOESM3_ESM.docx]

**Additional file 3**

| **Characteristics for patients without triage score** | |
| --- | --- |
| **Overall** | 5,892 |
| **Age groups** |  |
| 0-19 | 1317 (22,4) |
| 20-39 | 1412 (24,0) |
| 40-59 | 1354 (23,0) |
| 60-79 | 1213 (20,6) |
| >80 | 596 (10,1) |
| **Gender** |  |
| Female | 2797 (47,5) |
| Male | 3095 (52,5) |
| **CCI score** |  |
| Low (0) | 4659 (79,1) |
| Moderat (1-2) | 902 (15,3) |
| High (>=3) | 331 (5,6) |
| **Primary diagnosis** |  |
| Infectious diseases | 206 (3,5) |
| Neoplasm | 0 (0,0) |
| Hematological diseases | 17 (0,3) |
| Endocrine and nutritional diseases | 21 (0,4) |
| Mental and behavioral disorders | 26 (0,4) |
| Diseases of the nervous system | 51 (0,9) |
| Diseases of the circulatory system | 69 (1,2) |
| Diseases of the respiratory system | 6 (0,1) |
| Diseases of the digestive system | 45 (0,8) |
| Diseases of the musculoskeletal system | 90 (1,5) |
| Diseases of the genitourinary system | 72 (1,2) |
| Injury and poisoning | 1801 (30,6) |
| Factors influencing health status | 3145 (53,4) |
| Symptoms, signs, and abnormal findings | 300 (5,1) |
| Other | 43 (0,7) |
| **Admission time**  Weekday, daytime  Weekday, evening  Weekday, nighttime  Weekend, daytime  Weekend, evening  Weekend, nighttime | 2878 (48,9)  1213 (20,6)  225 (3,8)  536 (9,1)  790 (13,4)  250 (4,2) |
| **Length of stay within the hospital**  0-59 minutes (<1 hour)  60-179 minutes (1-2.59 hours)  180-259 minutes (3-5.59 hours)  360-719 minutes (6-11.59 hours)  720-1439 minutes (12-23.59 hours)  1440-2879 minutes (24-47.59 hours)  More than 2880 minutes (>48 hours) | 1293 (22,0)  1903 (32,3)  837 (14,2)  330 (5,6)  361 (6,1)  268 (4,6)  900 (15,3) |
| **Departments within the hospital**  Cardiology department  Neurological department  Other medical departments  Orthopedic surgery department  Other surgical departments  Gynecology department/obstetrics  Psychiatry  Pediatric ward  Another ED  ICU (intensive care unit)  **Departments at other hospitals** | 52 (3,6)  67 (4,7)  116 (8,1)  503 (35,1)  185 (12,9)  73 (5,1)  10 (0,7)  96 (6,7)  213 (14,9)  61 (4,3)  56 (3,9) |
